# Rasch Analysis of the Composite Autonomic Symptom Score (COMPASS-31) From Data Collected in an Online Population

**DOI:** 10.7759/cureus.108788

**Published:** 2026-05-13

**Authors:** Wim E Tuinebreijer, Henk M Koning

**Affiliations:** 1 Surgery, Erasmus University Medical Center, Rotterdam, NLD; 2 Anaesthesiology, Pain Clinic De Bilt, De Bilt, NLD

**Keywords:** autonomic nervous system, classical test theory, compass-31, composite autonomic symptom score, cronbach's alpha, internet panel, patient-reported outcome measure, rasch analysis, reliability, winsteps measurement software

## Abstract

Background: A Dutch internet panel with volunteers was assessed using the patient-reported outcome measure known as the Composite Autonomic Symptom Score (COMPASS-31).

Objectives: This study aimed to analyze the Dutch version of the COMPASS-31 using classical test theory and Rasch analysis, or one-parameter item response theory, to examine the questionnaire's structure for the second time in a different population. The first group consisted of clinical patients, while the second group consisted of the general population. The choice of the general population contrasts with most validation studies, which focus on clinical groups, as in our first study.

Methods: A transversal observational study of a cohort of 100 people voluntarily completed the Dutch version of the COMPASS-31 online. The sample population comprised 100 persons (42 females, 58 males). The age groups and their percentages were as follows: 18-29 years, 37%; 30-44 years, 22%; 45-60 years, 31%; and over 60 years, 10%. Classical test theory was performed after importing the data into IBM SPSS Statistics for Windows, Version 31.0 (IBM Corp., Armonk, New York, United States). Six Rasch analyses were completed for each domain of the COMPASS-31 using the Winsteps measurement software (Winsteps Rasch Measurement Version 5.10.0).

Results: The COMPASS-31 domains performed moderately in a group of adult volunteers. Cronbach's alpha across the domains was below 0.65, except for the Gastrointestinal, Bladder, and Pupillomotor domains, which were 0.65 or above. The person's reliability is below 0.8, the minimum level required for serious decision-making. Item reliability was good, except for the Bladder domain, which had a reliability of zero. Only the Pupillomotor domain can differentiate between the two population groups. Not all items in the Vasomotor and Gastrointestinal domains measure a single unidimensional variable, one of the prerequisites for a Rasch model. The probability curve for different domains performed adequately. Patients were able to distinguish between four levels of items. Some domains included categories that were too broad.

Conclusion: This study, conducted with a different group of internet volunteers, offered several valuable insights into the psychometric properties of the COMPASS-31 and its domains. Our data again did not align with the Rasch model. The analysis failed to confirm the scale's reliability, as the domains could differentiate only one or two levels of autonomic symptoms in adults. The person's reliability is below 0.8, which is the minimum threshold needed for meaningful decision-making. Only the Pupillomotor domain can distinguish between the two groups, while the other domains identify only one of them. The COMPASS-31 is a screening tool or descriptive profile, not a unidimensional measure designed to produce a single, interval-level score for any domain. Based on our findings, we recommend against using the total score as a single metric. The domains should serve as independent, ordinal indices. Item banks for computer adaptive testing (CAT) should be developed specifically for each domain.

## Introduction

Patient-reported outcome measures (PROMs) assess a patient's health status and therapy, as reported directly by patients without interpretation by a healthcare professional [1]. The Composite Autonomic Symptom Score (COMPASS-31) is an American English questionnaire that assesses autonomic nervous system symptoms across six domains with 31 items [2]. The six subclasses of the COMPASS-31 are as follows: Orthostatic Intolerance, Vasomotor, Secretomotor, Gastrointestinal, Bladder, and Pupillomotor. The COMPASS-31 has been translated into Italian [3,4], Croatian [5,6], Serbian [5,6], Indian [7], Korean [8], German [9], Norwegian [10], and Turkish [11]. Recently, the COMPASS-31 was translated into Dutch and tested for reliability and validity with a group of 62 chronic pain patients, using both classical test theory [12] and Rasch analysis (submitted for publication). Modern test theory offers many advantages, including a comprehensive examination of dimensionality, analysis of the data's fit to the Rasch model, and evaluation of category function [13,14]. The reliability of a scale depends on the sample of individuals being studied. Therefore, we evaluated the Dutch version of the COMPASS-31 with an online survey of 100 volunteers from the global Dutch panel on SurveyMonkey (https://www.surveymonkey.com). The objective of this study was to assess the reliability and validity of the COMPASS-31 in a volunteer group and compare the findings to those from a previous study involving chronic pain patients [12]. The first group consisted of clinical patients, while the second group consisted of the general population. The choice of the general population contrasts with most validation studies, which focus on clinical groups, as in our first study.

## Materials and methods

The study employed a cross-sectional observational cohort design. Dutch internet panelists from SurveyMonkey's global panel voluntarily completed the questionnaire over a two-day period. SurveyMonkey's global panelists are recognized for their high-quality respondents and engagement. Panelists are removed from the program when data are missing. Therefore, missing data were not a problem. The scoring algorithm was provided by Sletten et al. [2]. Responses are recorded on a scale of 2-6. After recording the item responses, scores for each domain are calculated to produce a final score using a scoring algorithm that ranges from 0 to 3. The final weighted domain scores vary from 5 to 40. A total COMPASS-31 score is obtained by summing the domain scores. No quality control procedures were applied during data collection from the online panel. An online population was selected to test the COMPASS-31 in a presumably healthier population. The sample included 100 participants (42 females, 58 males). The age distribution was as follows: 18-29 years, 37%; 30-44 years, 22%; 45-60 years, 31%; and over 60 years, 10%. This study was structured as a survey, and a sample size of about 100 was feasible within the available time and considered sufficient to collect meaningful data across different response categories [2]. However, it was at the lower end of acceptability, especially in domains with many items, such as the Gastrointestinal domain.

Statistical analysis

We imported the data into IBM SPSS Statistics for Windows, Version 31.0 (IBM Corp., Armonk, New York, United States), and analyzed it using classical test theory. We assessed the reliability of the COMPASS-31 test with Cronbach's alpha. We calculated the difficulty of each item by dividing the mean score by the maximum score. We evaluated item discrimination by measuring the correlation between individual item scores and the total scores of the domains. Rasch analysis was performed using Winsteps measurement software (Winsteps Rasch Measurement Version 5.10.0). A Partial Credit Model (PCM) was used because not all items had the same number of categories. We constructed the person-item map (also known as the Wright map) for each domain to illustrate the hierarchy of personal and item measures. How well the data fit or do not fit the model was tested (item fit statistics). Person and item reliability, as well as the separation coefficient, were estimated. The order of the categories was tested, and the dimensionality was investigated [13,14]. Because the COMPASS-31 is designed as a multi-domain profile and the total score is not unidimensional, Rasch analysis was conducted separately for each domain.

## Results

We present the details of the sample internet population and the former chronic pain population in Tables 1-2. The mean scores of the domains for the chronic pain patients and the volunteer internet group were similar. Only the Orthostatic Intolerance domain had a significantly higher mean in the volunteer internet group. Cronbach's alpha is a measure of reliability. Cronbach's alpha across the domains was 0.86 or lower. This is comparable to the chronic pain study, except for the Bladder domain, which has higher reliability (0.86 versus 0.69) in the volunteer group.

**Table 1:** Results of the analysis of the COMPASS-31 domains of the sample internet population COMPASS-31: Composite Autonomic Symptom Score

Statistics	Orthostatic Intolerance	Vasomotor	Secretomotor	Gastrointestinal	Bladder	Pupillomotor	Sum score
Mean score (SD)	15.6 (9.2)	1.06 (1.4)	4.5 (3.3)	6.9 (4.5)	1.7 (2.4)	1.6 (1.1)	31.2 (16.6)
Cronbach's alpha	0.49	0.21	0.57	0.65	0.86	0.71	0.70

**Table 2:** Results of the analysis of the COMPASS-31 domains in chronic pain patients. Former study for comparison. Independent t-test, comparing the mean score of the domains of the sample internet population with those of the former chronic pain population COMPASS-31: Composite Autonomic Symptom Score

Statistics	Orthostatic Intolerance	Vasomotor	Secretomotor	Gastrointestinal	Bladder	Pupillomotor	Sum score
Mean score (SD)	12.4 (10.4)	0.71 (1.2)	3.1 (3.7)	6.1 (5.2)	1.7 (2.0)	1.8 (1.4)	25.7 (16.4)
Independent t-test	0.04	0.12	0.10	0.30	0.99	0.22	0.04

The calculated difficulty levels of the items are shown in Table 3. No significant changes are observed, except that the most difficult to endorse item in the Gastrointestinal domain is now item 18 ("How severe are these bouts of diarrhea?"). It was previously item 22, which concerns the severity of episodes of constipation. Items that ask about severity and changes over time tend to be more difficult.

**Table 3:** Difficulty of the items of the COMPASS-31 COMPASS-31: Composite Autonomic Symptom Score

Item	Items	N	Maximum score	Mean	Item difficulty
Orthostatic Intolerance 1	1	100	1	0.76	0.76
Orthostatic Intolerance 2	2	81	3	0.95	0.32
Orthostatic Intolerance 3	3	83	3	1.59	0.53
Orthostatic Intolerance 4	4	83	3	1.28	0.43
Vasomotor 1	5	100	1	0.32	0.32
Vasomotor 2	6	38	1	1.00	1.00
Vasomotor 3	7	39	3	1.46	0.49
Secretomotor 1	8	99	2	0.23	0.12
Secretomotor 2	9	100	1	0.39	0.39
Secretomotor 3	10	100	1	0.41	0.41
Secretomotor 4	11	99	3	1.10	0.37
Gastrointestinal 1	12	100	2	0.54	0.27
Gastrointestinal 2	13	100	2	0.93	0.465
Gastrointestinal 3	14	99	2	0.28	0.14
Gastrointestinal 4	15	100	2	0.74	0.37
Gastrointestinal 5	16	100	1	0.54	0.54
Gastrointestinal 6	17	61	3	0.72	0.24
Gastrointestinal 7	18	60	2	1.57	0.785
Gastrointestinal 8	19	62	3	1.10	0.367
Gastrointestinal 9	20	100	1	0.42	0.42
Gastrointestinal 10	21	57	3	0.96	0.32
Gastrointestinal 11	22	57	3	1.65	0.55
Gastrointestinal 12	23	57	3	1.26	0.42
Bladder 1	24	98	3	0.49	0.163
Bladder 2	25	99	3	0.48	0.16
Bladder 3	26	99	3	0.53	0.177
Pupillomotor 1	27	98	3	0.80	0.266
Pupillomotor 2	28	58	3	1.76	0.587
Pupillomotor 3	29	98	3	0.83	0.277
Pupillomotor 4	30	63	3	1.59	0.53
Pupillomotor 5	31	99	3	1.15	0.383

The item discriminations, shown as correlations, are listed in Table 4. These correlations are positive and significant, similar to what we observed in our chronic pain patients.

**Table 4:** Item discrimination of the COMPASS-31 *Cannot be computed because at least one of the variables is constant. COMPASS-31: Composite Autonomic Symptom Score

Item	Items	N	Pearson correlation	Sig. (two-tailed)
Orthostatic Intolerance 1	1	100	0.75	<0.0001
Orthostatic Intolerance 2	2	81	0.70	<0.0001
Orthostatic Intolerance 3	3	83	0.77	<0.0001
Orthostatic Intolerance 4	4	83	0.66	<0.0001
Vasomotor 1	5	100	0.91	<0.0001
Vasomotor 2	6	38	a*	-
Vasomotor 3	7	39	0.91	<0.0001
Secretomotor 1	8	99	0.49	<0.0001
Secretomotor 2	9	100	0.66	<0.0001
Secretomotor 3	10	100	0.68	<0.0001
Secretomotor 4	11	99	0.81	<0.0001
Gastrointestinal 1	12	100	0.40	<0.0001
Gastrointestinal 2	13	100	0.36	<0.0001
Gastrointestinal 3	14	99	0.47	<0.0001
Gastrointestinal 4	15	100	0.61	<0.0001
Gastrointestinal 5	16	100	0.51	<0.0001
Gastrointestinal 6	17	61	0.27	<0.0001
Gastrointestinal 7	18	60	0.41	<0.0001
Gastrointestinal 8	19	62	0.53	<0.0001
Gastrointestinal 9	20	100	0.56	<0.0001
Gastrointestinal 10	21	57	0.59	0.01
Gastrointestinal 11	22	57	0.54	<0.0001
Gastrointestinal 12	23	57	0.48	<0.0001
Bladder 1	24	98	0.88	<0.0001
Bladder 2	25	99	0.89	<0.0001
Bladder 3	26	99	0.89	<0.0001
Pupillomotor 1	27	98	0.74	<0.0001
Pupillomotor 2	28	58	0.58	<0.0001
Pupillomotor 3	29	98	0.73	<0.0001
Pupillomotor 4	30	63	0.75	<0.0001
Pupillomotor 5	31	99	0.68	<0.0001

In Table 5, we present the score and measure order of the domain items in logit units.

**Table 5:** Item statistics of the domains Reasonable ranges of infit and outfit mean squares for interpretation are between 0.5 and 1.5.

Domain	Items	Count	Measure	Infit mean square	Outfit mean square
Orthostatic Intolerance	1. In the past year, have you ever felt faint, dizzy, or "goofy" or had difficulty thinking soon after standing up from a sitting or lying position?	76	1.18	0.64	0.55
	2. When standing up, how frequently do you get these feelings or symptoms?	77	1.03	1.40	1.45
	4. In the past year, have these feelings or symptoms that you have experienced:	106	-0.52	1.42	1.26
	(1) Gotten much worse. (2) Gotten somewhat worse. (3) Stayed about the same. (4) Gotten somewhat better. (5) Gotten much better. (6) Completely gone				
	3. How would you rate the severity of these feelings or symptoms?	132	-1.70	0.56	0.52
Vasomotor	5. In the past year, have you ever noticed color changes in your skin, such as red, white, or purple?	32	2.05	0.91	0.67
	6. Which parts of your body are affected by these color changes?	38	0.33	0.46	0.27
	7. Are these changes in your skin color:	57	-2.38	0.67	3.27
	(1) Getting much worse. (2) Getting somewhat worse. (3) Staying about the same. (4) Getting somewhat better. (5) Getting much better. (6) Completely gone				
Secretomotor	8. In the past 5 years, what changes, if any, have occurred in your general body sweating?	23	1.83	1.11	1.68
	9. Do your eyes feel excessively dry?	39	0.70	0.92	0.86
	10. Does your mouth feel excessively dry?	41	0.57	0.81	0.77
	11. For the symptom of dry eyes or dry mouth that you have had for the longest period of time, is this symptom:	109	-3.09	1.00	1.03
	(1) I have not had any of these symptoms. (2) Getting much worse. (3) Getting somewhat worse. (4) Staying about the same. (5) Getting somewhat better. (6) Getting much better. (7) Completely gone				
Gastrointestinal	14. In the past year, have you vomited after a meal?	28	2.00	1.06	0.97
	20. In the past year, have you been constipated?	42	1.33	0.86	0.84
	12. In the past year, have you noticed any changes in how quickly you get full when eating a meal?	54	0.84	1.30	1.22
	16. In the past year, have you had any bouts of diarrhea?	54	0.84	0.85	0.92
	17. How frequently does this occur?	44	0.61	1.22	1.17
	15. In the past year, have you had cramping or colicky abdominal pain?	74	0.13	0.96	0.93
	21. How frequently are you constipated?	55	-0.09	1.25	1.20
	13. In the past year, have you felt excessively full or persistently full (bloated feeling) after a meal?	93	-0.48	0.84	0.85
	19. Are your bouts of diarrhea:	68	-0.52	1.33	1.33
	(1) Getting much worse. (2) Getting somewhat worse. (3) Staying about the same. (4) Getting somewhat better. (5) Getting much better. (6) Completely gone				
	23. Is your constipation:	72	-0.93	1.38	1.34
	(1) Getting much worse. (2) Getting somewhat worse. (3) Staying about the same. (4) Getting somewhat better. (5) Getting much better. (6) Completely gone				
	18. How severe are these bouts of diarrhea?	94	-1.79	0.52	0.52
	22. How severe are these episodes of constipation?	94	-1.95	0.63	0.63
Bladder	25. In the past year, have you had difficulty passing urine?	48	0.10	1.03	0.98
	24. In the past year, have you ever lost control of your bladder function?	48	0.10	0.90	0.92
	26. In the past year, have you had trouble completely emptying your bladder?	52	-0.20	1.04	1.11
Pupillomotor	27. In the past year, without sunglasses or tinted glasses, has bright light bothered your eyes?	78	1.02	1.41	1.34
	29. In the past year, have you had trouble focusing your eyes?	81	0.90	1.17	1.11
	31. Is the most troublesome symptom with your eyes (i.e., sensitivity to bright light or trouble focusing):	114	-0.28	1.11	1.14
	(1) I have not had any of these symptoms. (2) Getting much worse. (3) Getting somewhat worse. (4) Staying about the same. (5) Getting somewhat better. (6) Getting much better. (7) Completely gone				
	30. How severe is this focusing problem?	100	-0.62	0.49	0.48
	28. How severe is this sensitivity to bright light?	102	-1.02	0.60	0.64

From the Orthostatic Intolerance domain, item 1 remains the most difficult to endorse. Item 2 has almost the same level of difficulty and swapped places with item 4 in the chronic pain patient's study [12]. The infit and outfit mean squares were between 0.5 and 0.7 (reasonable ranges for clinical observations).

Item 5 of the Vasomotor domain is the most difficult to endorse. Item 6 is less difficult and has replaced item 5 in studies involving patients with chronic pain [12]. Item 5 had low infit mean squares (0.46), which may cause misleadingly high reliability due to over-predictability. Item 6 had high outfit mean squares (3.27), indicating distortion in the measurement system. Item 6 has two answers, both coded with the same count (1).

Item 8 of the Secretomotor domain is the most difficult to endorse. Items 9 and 10 are less difficult and have been swapped with item 8 in the study with patients experiencing chronic pain [12].

Item 14 in the Gastrointestinal domain represents the highest level of the latent trait. The first four items had the same order as the items in the study with patients experiencing chronic pain.

The top items of the Bladder domain represent the highest level of the latent trait. Items 25 and 24 are more difficult to endorse than item 26. These had the same order as the items in the study with patients experiencing chronic pain [12].

Item 27 of the Pupillomotor domain was the most difficult to endorse and swapped places with item 29 [12]. Item 31 had low infit mean squares (0.49) and low outfit mean squares (0.48). This misfitting item was asking for a change in a symptom over time, and the answers were too predictable.

Table 6 shows the reliability of the six domains. In both studies, the person's reliability was below 0.8, which is the minimum level needed for serious decision-making. Item reliability was good compared to the chronic pain study, likely due to the larger sample size. The item reliability of the bladder domain was 0.00, due to low variability, as nearly all respondents gave the same answer. The person separation was low except for the Pupillomotor domain, which resulted in a strata index of 1 for all domains except Pupillomotor, which had a separation index of 2 [15,16]. A strata index below 2 indicates that the scale can distinguish fewer than 3 distinct strata or groups of person ability. Only one group of people in the Orthostatic Intolerance, Vasomotor, Secretomotor, Gastrointestinal, and Bladder domains could be distinguished. People in the Pupillomotor domain could be separated into two groups.

**Table 6:** Reliability of the Orthostatic Intolerance, Vasomotor, Secretomotor, Gastrointestinal, Bladder, and Pupillometer domains

Coefficients	Orthostatic Intolerance	Vasomotor	Secretomotor	Gastrointestinal	Bladder	Pupillomotor
Item reliability	0.95	0.93	0.98	0.96	0.00	0.93
Item separation	4.50	3.70	7.14	5.06	0.00	3.65
Person reliability	0.29	0.29	0.19	0.57	0.39	0.61
Person separation	0.64	0.64	0.48	1.16	0.80	1.26
Strata index	1.19	1.19	0.97	1.4	0.97	2.01

Summaries of category structures for various domains are shown in Tables 7-12 [17,18].

**Table 7:** Summary of the category structure of the Orthostatic Intolerance domain

Category label/score	Observed count	Observed count %	Observed average	Outfit mean square	Threshold
0	54	16	-3.29	1.13	None
1	210	61	-1.43	0.88	-4.22
2	68	20	1.15	0.57	0.97
3	15	4	2.43	1.59	3.25

**Table 8:** Summary of the category structure of the Vasomotor domain

Category label/score	Observed count	Observed count %	Observed average	Outfit mean square	Threshold
0	72	41	-6.40	3.07	None
1	89	50	-1.98	0.91	-6.98
2	10	6	2.99	0.33	2.95
3	6	3	4.74	0.27	4.04

**Table 9:** Summary of the category structure of the Secretomotor domain

Category label/score	Observed count	Observed count %	Observed average	Outfit mean square	Threshold
0	223	56	-5.04	1.17	None
1	142	36	-2.63	1.20	-3.73
2	29	7	0.76	0.59	0.66
3	4	1	2.06	0.61	3.07

**Table 10:** Summary of the category structure of the Gastrointestinal domain

Category label/score	Observed count	Observed count %	Observed average	Outfit mean square	Threshold
0	360	38	-3.11	1.03	None
1	432	45	-1.67	0.89	-2.65
2	143	15	-0.08	0.91	0.19
3	18	2	0.50	1.40	2.46

**Table 11:** Summary of the category structure of the Bladder domain

Category label/score	Observed count	Observed count %	Observed average	Outfit mean square	Threshold
0	190	64	-3.02	0.99	None
1	78	26	-1.43	1.01	-2.96
2	14	5	0.43	0.69	1.20
3	14	5	0.94	1.49	1.76

**Table 12:** Summary of the category structure of the Pupillomotor domain

Category label/score	Observed count	Observed count %	Observed average	Outfit mean square	Threshold
0	106	25	-2.50	1.11	None
1	173	42	-1.21	0.93	-2.82
2	109	26	0.57	0.80	0.08
3	28	7	2.22	1.24	2.73

In the Orthostatic Intolerance domain, the difference between thresholds 2 and 1 exceeded five log units. Most patients (61%) responded with this score. This pattern was also observed in a study of chronic pain patients [12].

The Vasomotor domain categories featured a very broad first category (9.93 logits) and a relatively narrow second category (1.09 logits). The zero category had a high mean square of outfit at 3.07. The third category was rare, with only six patients. Similar patterns were seen in the chronic pain study.

In the Secretomotor domain, categories were ordered, and only four individuals (1%) selected the last category. The outfit mean squares for these categories were all below 2.

The category structures of the Gastrointestinal domain were organized. The outfit mean squares were all below 2. Only the last (third) category was small (1.4 logits broad).

The category structure of the Bladder domain showed all outfit mean squares below 2. Only category 2 was small (0.58 logits). In the chronic pain study, thresholds 2 and 3 were disordered [12].

The Pupillomotor domain thresholds were ordered and showed no misfit. This pattern was also seen in the study of chronic pain patients [12].

In Table 13, we present the results of the dimensionality analysis. Rasch factor analysis examines the residuals remaining after extracting the linear Rasch measure from the dataset. A secondary dimension in the data must account for the variance of at least two items. Eigenvalue units represent the residual or unexplained variance. The eigenvalue indicates the number of items that are off-dimension. The Vasomotor and Gastrointestinal domain items showed about two eigenvalue units of unexplained variance. These two domains were also observed in the study of chronic pain patients. In the Vasomotor domain, the first factor involves changes in skin color and their location, compared to the second factor, which is skin color changes over time. In the Gastrointestinal domain, the first factor focused on changes in bouts of diarrhea and constipation over the past year, as well as the frequency of constipation, compared to the second factor, which included abdominal pain, the frequency of diarrhea, and the severity of diarrhea.

**Table 13:** Principal component analysis of the residuals of domain items

Items	Items	Raw variance explained %	Unexplained variance first contrast (eigenvalue units)	Items first contrast	Items second contrast
Orthostatic Intolerance domain	4	51.7	1.67	-	-
Vasomotor domain	3	76.4	1.98	Items 5 and 6	Item 7
Secretomotor domain	4	59.4	1.65	-	-
Gastrointestinal domain	12	45.6	1.96	Items 19, 20, and 21	Items 15, 17, and 18
Bladder domain	3	45.5	1.63	-	-
Pupillomotor domain	5	53.1	1.75	-	-

In Table 14, we display the results of independent t-tests comparing the means of different COMPASS-31 domains for females and males in the computer survey and the chronic pain patient study [12]. Significant differences were observed between females and males in the Secretomotor, Gastrointestinal, and Pupillomotor domains among chronic pain patients, but not among computer survey participants [12].

**Table 14:** COMPASS-31 domain scores for different genders in different groups of people COMPASS-31: Composite Autonomic Symptom Score

Computer survey people (N=100)				Chronic pain patients (former study) [3]		
Domains	Female, mean (SD)	Male, mean (SD)	P-value t-test	Female, mean (SD)	Male, mean (SD)	P-value t-test
Orthostatic Intolerance domain	16.6 (8.7)	15.0 (9.6)	0.39	19.0 (6.7)	18.3 (6.5)	0.71
Vasomotor domain	0.73 (1.3)	1.3 (1.5)	0.06	2.6 (0.78)	2.5 (0.83)	0.75
Secretomotor domain	4.6 (3.8)	4.5 (3.0)	0.80	4.0 (3.8)	1.8 (3.2)	0.02
Gastrointestinal domain	7.3 (4.4)	6.6 (4.5)	0.46	7.3 (5.3)	4.4 (4.8)	0.03
Bladder domain	1.4 (2.0)	1.9 (2.6)	0.30	1.6 (2.1)	1.8 (2.0)	0.62
Pupillomotor domain	1.5 (1.2)	1.6 (1.1)	0.54	2.2 (1.4)	1.4 (1.4)	0.02

## Discussion

This is our second study using the Rasch model to analyze the COMPASS-31. In our previous research, we examined a group of 62 chronic pain patients [12]. In this study, we analyzed data from 100 individuals who voluntarily participated in a survey on SurveyMonkey Europe (https://www.surveymonkey.com).

Person reliability was low across most domains, except for the Pupillomotor domain. Strata indices for the domains were around 1, except for the Pupillomotor domain. As a result, only single-person groups could be distinguished from all domains, except for the two-person groups in the Pupillomotor domain.

Rasch analysis showed no significant changes in item order across the domains in both studies [12]. Category structures were disorganized, displaying excessively wide and narrow thresholds in the Orthostatic Intolerance, Vasomotor, and Bladder domains in both studies [12]. Dimensionality analysis using Rasch factor analysis indicated that the Vasomotor and Gastrointestinal domain items had two eigenvalues of unexplained variance, suggesting the potential presence of a second dimension in the data. Multidimensionality violates the Rasch model. Low person reliability and separation indicate that the COMPASS-31 cannot reliably distinguish among different levels of symptom severity in the population. Rasch violations of the domains are presented in Table 15 [15].

**Table 15:** COMPASS-31 domains with Rasch violations +: present; -: absent COMPASS-31: Composite Autonomic Symptom Score

Domains	Low person reliability	Disordered categories	Multidimensionality	Local dependency
Orthostatic Intolerance domain	+	+	-	+
Vasomotor domain	+	+	+	+
Secretomotor domain	+	-	-	-
Gastrointestinal domain	+	-	+	+
Bladder domain	+	+	-	+
Pupillomotor domain	-	-	-	+

Classical test theory showed the following results. The reliability in both studies was moderate, with Cronbach's alphas ranging from 0 to 0.70 in the chronic pain group and from 0.21 to 0.86 in this online survey. Cronbach's alphas for the Orthostatic Intolerance, Vasomotor, and Secretomotor domains in both studies were 0.60 or below. Item difficulty and discrimination showed no significant changes across the two studies [12]. 

In the study of chronic pain patients [12], a significant difference was found in the means of the Secretomotor, Gastrointestinal, and Pupillomotor domains between female and male patients. Females had higher scores in these three domains. These significant differences between females and males for the Secretomotor and Gastrointestinal domains, but not the Pupillomotor domain, were also observed in a study of patients with longstanding type 2 diabetes [8]. This difference in the Pupillomotor domain can be attributed to the differences between the patient groups: chronic pain versus type 2 diabetes. In our current study, which employed an internet survey, no significant difference was found between female and male patients, likely because few individuals exhibited autonomic symptoms, as indicated by the low mean sum scores in the relevant domains. This floor effect compresses scores and contributes to low person separation. 

This study has several limitations, particularly the moderate number of patients and the online recruitment method. The online volunteer population may not accurately represent the general or clinical populations. We suggest conducting a prospective study with a larger patient cohort and evaluating patients both before and after therapy. The absence of clinical data on participants' health status is a significant limitation, as it is unknown whether they have conditions associated with dysautonomia. Our sample size of 100 is on the lower end of acceptability, especially for domains with many items, such as the Gastrointestinal domain. Low symptom prevalence and limited variability may have influenced reliability and dimensionality findings.

## Conclusions

This study, which used internet data, did not fit the Rasch model, consistent with our previous study involving chronic pain patients. The items show dependency. This indicates that responses to some items are correlated beyond what is expected from the underlying trait, often because they are phrased similarly or are conditional; the answer to a specific item influences which items are answered next. The reliability is too low. Only one group from different domains, except the Pupillomotor domain, could be distinguished. The COMPASS-31 total score is multidimensional; therefore, we examined its domains. Even the Vasomotor and Gastrointestinal domains are likely still multidimensional and should be further analyzed. This analysis of the Vasomotor and Gastrointestinal domains could involve testing a bifactor model, combining or removing items to achieve unidimensionality, or conducting a qualitative study to assess whether the items are interpreted as intended by respondents. The COMPASS-31 is a screening tool or descriptive profile, not a unidimensional measure designed to produce a single, interval-level score for any domain. Based on our findings, we recommend against using the total score as a single metric. The domains should serve as independent, ordinal indices. Item banks for computer adaptive testing (CAT) should be developed specifically for each domain. These conclusions should be accepted with caution due to the limited number of patients and the online recruitment of patients.
